# Esophageal Adenocarcinoma in the Proximal Esophageal Segment: A Unique Presentation in a Male With Alcohol Abuse

**DOI:** 10.7759/cureus.8863

**Published:** 2020-06-27

**Authors:** Brandon S Shiflett, Lakmal S Ekanayake, Anastacia L Rodriguez, Ilyas Ikramuddin, Carla Myers

**Affiliations:** 1 Internal Medicine, Grandview Medical Center, Dayton, USA; 2 Medicine, Ohio University Heritage College of Osteopathic Medicine, Athens, USA; 3 Internal Medicine: Gastroenterology, Dayton Gastroenterology Inc., Dayton, USA

**Keywords:** esophageal adenocarcinoma

## Abstract

Esophageal adenocarcinoma (EAC) is a malignancy classically seen in the distal esophagus. While many risk factors associated with the condition have been reported, the most common among them are gastroesophageal reflux disease (GERD) and obesity. Histological changes range from metaplasia within the esophagus from stratified squamous epithelium to non-ciliated columnar cells with goblet cells. In contrast, squamous cell carcinoma (SCC) is classically found in the proximal portion of the esophagus and its risk factors include tobacco and alcohol use. We present a unique case of a 59-year-old African American male who presented to the ED with dysphagia, weight loss, and multiple episodes of emesis. Notable medical history included tobacco abuse, alcohol abuse, and alcoholic cirrhosis. Currently, there are numerous case reports delineating unique presentations of esophageal cancers; however, there are few case reports that demonstrate EAC affecting the proximal segment of the esophagus.

## Introduction

Esophageal cancer can be classified into two types: esophageal adenocarcinoma (EAC) and squamous cell carcinoma (SCC). EAC is a form of esophageal cancer that arises from histological changes of the normal esophageal mucosa [1]. EAC originates from metaplasia of the epithelial cells of the esophagus [2]. Significantly, this can transition into dysplasia of mucosa, which often represents malignancy.

A subset of the pathologies that arise from sustained and untreated gastroesophageal reflux disease (GERD) can lead to Barrett’s esophagus, esophageal strictures, and fibrous rings [3]. The disease pathogenesis of EAC mainly occurs within the lower one-third of the esophagus near the gastroesophageal junction [4]. Histologically, we can see an increase in the amount of mucin and mucin-secreting glands [5]. The incidence of EAC is seven times more common in men than in women [5]. In recent years, the incidence of EAC has increased in the Western world [6]. 

SCC represents a malignant transformation of the epithelial that occurs in the upper or middle third of the esophagus. Key risk factors for SCC include alcohol and tobacco abuse and previous radiation exposure [7]. Geographically, SCC is most commonly found in Asia, Africa, and South America, and these regions also account for 90% of all esophageal cancer cases worldwide [8]. SCC is a disease commonly associated with middle-aged adults, and it is six times more common in African Americans compared to other races and ethnicities [9].

In this report, we discuss a case of an atypical presentation of EAC with a unique anatomic location of an esophageal mass. To the best of our knowledge, there are a very limited number of case reports that demonstrate EAC found within the proximal segment of the esophagus.

## Case presentation

A 59-year-old African American male presented to the ED with the chief complaint(s) of nausea, vomiting, abdominal pain, and chest pain for two weeks. He reported significant weight loss of two to five kilograms, night sweats, aching abdominal pain, and an intense nauseated feeling upon the ingestion of solids and liquids. Additional complaints included dysphagia and globus sensation. Medical history included tobacco and alcohol abuse, alcoholic cirrhosis status-post transjugular intrahepatic portosystemic shunt (TIPS) procedure in 2014, myocardial infarction, and pancreatitis.

In the ED, the patient was started on ondansetron, pantoprazole, and intravenous fluids. Physical examination and vitals were within normal limits. Electrocardiogram showed sinus rhythm, multiple prolonged QT interval, multiple premature ventricular contractions; routine blood work was also performed (Table 1). Due to the patient’s intractable nausea and worrisome symptoms of weight loss and dysphagia, he was hospitalized. Gastroenterology consultation was made and, subsequently, an esophagogastroduodenoscopy (EGD) was performed.

**Figure 1:**
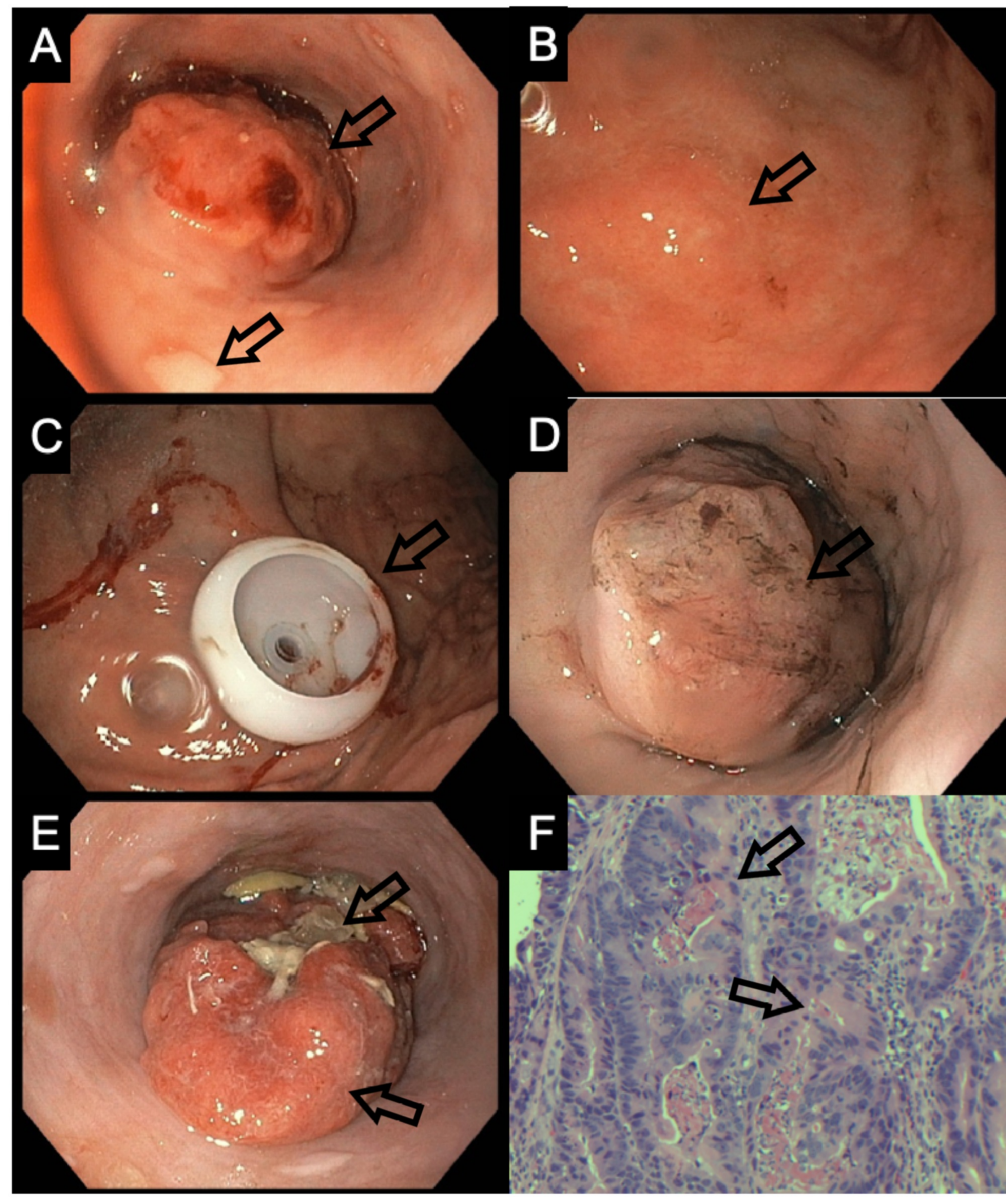
EGD findings A: mass on initial EGD; B: continuation of mass on initial EGD; C: PEG placement; D: mass on first readmission EGD; E: mass on second readmission EGD with impaction; F: histology of initial biopsy EGD: esophagogastroduodenoscopy; PEG: percutaneous endoscopic gastrostomy

Two weeks after discharge from the hospital, the patient returned for a follow-up. A repeat EGD was performed. Results showed an esophageal obstruction secondary to esophageal cancer with food impaction and hiatal hernia (Figure 1E).

**Table 1:** Significant laboratory values upon patient presentation in ED AST: aspartate aminotransferase; ED: emergency department

	Values	Reference range
Red blood cells, M/uL	4.09	4.30-5.86
Platelets, K/uL	115	154-393
Albumin, g/dL	2.2	3.4-5.0
Alkaline phosphate, U/L	127	45-117
AST, U/L	100	15-37
Total bilirubin, MG/DL	1.9	0.2-1.0
Direct bilirubin, MG/DL	1.10	0.00-0.20

EGD was significant for a malignant-appearing esophageal mass of 28 centimeters (Figure 1A-1E). This mass was present throughout a portion of the proximal third and entire middle third of the esophagus. No mucosal abnormalities were present at the gastroesophageal junction. During the biopsy, the mass was noted to be hard and friable. In addition, a hiatal hernia was observed on CT. Based on the location of the mass and the patient's history of alcohol and tobacco use, it was assumed to be SCC. Pathology of the mass showed moderately differentiated invasive adenocarcinoma (Figure 1F). The proximal location of the mass is an atypical presentation of EAC. After percutaneous endoscopic gastrostomy (PEG) tube placement (Figure 1C), the patient was discharged in stable condition and instructed to follow up on his progress.

## Discussion

The etiologies of EAC are well known, with obesity and long-standing GERD being the most common risk factors associated with the condition. Reflux disease affects as many as one in five men and women in the United States (US) [10]. In 2009, gastrointestinal diseases of all types cost the US approximately $142 billion in healthcare expenses, with GERD accounting for $20 billion of it [10]. The ability of a physician to identify significant risk factors from a patient’s history is pertinent for image modality selection and appropriate diagnosis. A differential diagnosis based on signs and symptoms of dysphagia, multiple episodes of nausea, and vomiting must include malignancy, mechanical obstruction, and atypical anatomical differences.

This case highlights the importance of identifying key risk factors through a complete history and physical while keeping a broad differential diagnosis. The patient presented with a history of alcohol abuse, alcoholic cirrhosis, and an extensive smoking history. Presenting symptoms of weight loss, dysphagia, and night sweats led us to the possibility of malignancy. EGD showed a large mass in the proximal and middle third of the esophagus. This case presented symptomatically and historically as a squamous cell esophageal carcinoma; however, biopsy demonstrated a moderately differentiated invasive EAC. Of note, EAC is one of the most common malignant esophageal tumors. Increased incidences of EAC are found in those with GERD, smoking, and obesity. Individuals with esophageal cancer present with symptoms similar to those of dysphagia, odynophagia, weight loss, and more non-specific symptoms like cough, dyspnea, and hoarseness.

In our case presentation, we described a patient presenting with atypical, moderately differentiated invasive EAC with a history of alcohol abuse and alcoholic cirrhosis. The existing literature does not contain much information on EAC tumors presenting in the proximal third of the esophagus. Hence, more studies are needed to further investigate the incidence, risks, morbidity, and mortality related to atypically presenting EACs in the proximal portion of the esophagus.

## Conclusions

EAC is a major medical condition that presents as a malignant disease process. As clinicians, we must be aware that some patients, such as the one described above, may not adhere to hallmark risk factors; lesser-known predispositions may synergize to develop into EAC. Currently, the standard treatment for EAC is chemoradiation and surgery. We must always recognize the importance of identifying key risk factors for EAC, which will aid in early diagnostic testing and appropriate care for the patients. In our case, being keen clinicians who were willing to investigate beyond the presenting symptoms was what ultimately aided in diagnosing the specific type of esophageal cancer in our patient and thereby determining the optimal treatment strategy.
